# Inhibiting endoplasmic reticulum stress and unfolded protein response: an effective strategy for chemoprevention of hepatocarcinogenesis

**DOI:** 10.1007/s11010-026-05708-6

**Published:** 2026-08-24

**Authors:** Maya P. Shetty, Smita Hegde, Komal Rana, Sanjay Bharati

**Affiliations:** 1 Department of Nuclear Medicine, Manipal College of Health Professions, Manipal Academy of Higher Education , Manipal, India; 2 Advanced Research Centre, Manipal – Government of Karnataka Bioincubator, Manipal Academy of Higher Education, Manipal, India

**Keywords:** Hepatocellular carcinoma, Endoplasmic reticulum stress, GRP78, Unfolded protein response, ER stress inhibitor, BiP inducer X, Carcinogenesis

## Abstract

Endoplasmic reticulum stress and activation of the unfolded protein response are critical contributors to hepatocarcinogenesis. Targeting these pathways represents a novel strategy for liver cancer prevention. In this study, a hepatocarcinogenesis model was developed by administering *Diethylnitrosamine (DEN)* (50 mg/kg b.w, i.p) to male Wistar rats. The endogenous chaperone-inducer BIX was administered (0.1 mg/kg, bw, i.p) for the prevention of *DEN-*induced hepatocarcinogenesis. After 20 weeks of BIX treatment, its effect on tumor growth was assessed in terms of tumor statistics, tumor-dielectric properties and histopathology. The ER-stress inhibition potential of BIX was assessed using UPR-related genes and protein expression. The inflammatory, proliferative and apoptotic markers were assessed using enzyme immunoassay and fluorescent immunohistochemistry. BIX treatment impeded the growth of hepatic tumors, as demonstrated by decreased total number of tumors and tumor multiplicity. Histopathologically, the tumors in the BIX-protected group had lower histological grading as compared to untreated tumors. This was further supported by dielectric assessment of tumors, suggesting suppressed tumor development. BIX treatment significantly decreased the expression of UPR genes (*PERK*, *ATF-6*, *CHOP*) and proteins (p-PERK/PERK), respectively. Further, significantly decreased expression of inflammatory, proliferative and increased apoptotic markers confirmed the effectiveness of BIX in preventing ER stress-induced hepatocarcinogenesis. BIX effectively attenuated UPR activation and impeded tumor growth.

**Supplementary Information:**

The online version contains supplementary material available at https://doi.org/10.1007/s11010-026-05708-6.

## Introduction

Endoplasmic Reticulum (ER) serves as a critical organelle in maintaining cellular equilibrium by ensuring proper protein folding, post-translational modifications, and trafficking [1]. However, various physiological and pathological insults, including oxidative stress, nutrient deprivation, hypoxia, imbalanced intracellular Ca^2+^ and oncogene activation, can disrupt ER protein homeostasis [2]. Disruption of ER activity results in the aggregation of misfolded proteins within the ER lumen, leading to ER stress [3]. When misfolded proteins accumulate beyond the ER’s folding capacity, cells activate a highly conserved intracellular transduction pathway known as the unfolded protein response (UPR) [4]. UPR is basically an adaptive response that is governed by ER-localized transmembrane sensors PERK, IRE1α, and ATF6 [5]. It has been well documented that transmembrane sensors remain inactive during normal conditions due to their association with a molecular chaperone known as glucose-regulating protein (GRP78/BiP) [6]. However, upon ER stress, GRP78/BiP, selectively attaches to hydrophobic regions exposed on misfolded or unfolded proteins, resulting in their disengagement from ER luminal regions of PERK, IRE1α and ATF6 [7]. This dissociation leads to activation of PERK, IRE1α and ATF-6, which collectively function to reduce the load of misfolded proteins, thereby restoring ER homeostasis [8]. However, if UPR fails to overcome ER stress cells immediately trigger apoptosis [9].

Although the activation of UPR initially serves as a compensatory mechanism to re-establish ER homeostasis, its sustained engagement due to low-level and prolonged ER activation might paradoxically contribute to the onset of carcinogenesis [10]. A key factor for this switch from adaptation to pathology might be due to the imbalance between pro-survival and pro-apoptotic signals in the UPR network [11, 12]. The prolonged activation of PERK-eIF2α, IRE1α–XBP1s and ATF-6 pathway as observed in cancer cells, leads to the induction of antioxidant responses, continued proliferation, angiogenesis, resistance to apoptosis and enables cells to survive under stress [13, 14]. Thus, reducing the burden of ER and preventing the accumulation of misfolded proteins can be a potential strategy to inhibit tumorigenesis.

GRP78 is a key resident molecular chaperone of the endoplasmic reticulum (ER) with the ability to mitigate ER stress [7]. Thus, targeted upregulation of GRP78 without triggering the activation of UPR can be a promising strategy to prevent the accumulation of misfolded proteins that subsequently leads to alleviation of ER stress [15]. This approach has been widely explored by several research groups in various other pathological conditions, including diabetes, retinal neurodegeneration, stroke, and renal ischemia-reperfusion injury [16–19]. However, the potential of endogenous upregulation of GRP78/BiP to inhibit ER stress during early oncogenic transformation and its effect on the development of tumors has not been explored so far.

Therefore, in the present study, we aimed to evaluate the chemopreventive potential of an endogenous chaperone inducer, 1-(3,4-dihydroxy phenyl)-2-thiocyanato-ethanone (BIX), in preventing *Diethylnitrosamine (DEN)-*induced hepatocarcinogenesis.

## Materials and methods

### Chemical reagents and testing kits

1-(3,4-dihydroxyphenyl)-2-thiocyanato-ethanone (BIX, Sigma, SML1073), *Diethylnitrosamine (DEN)*, DCFH-DA and thiobarbituric acid were supplied by Sigma-Aldrich Co. (Missouri, US). Dimethyl sulfoxide was purchased from HiMedia Laboratories (Mumbai, India). Trizol, K0221 Maxima SYBR Green/ROX qPCR Master Mix. Antibodies GRP-78 (MA5-27686, RRID: AB_2735208), PERK (PERK-101AP, RRID: AB_2746309), p-PERK (MA5-15033, RRID: AB_10980432), Ki-67 (MA5-14520, RRID: AB_10979488), IL-6 (BS0782R, RRID: AB_10859871), IL-10 (PA5-85660, RRID: AB_2792799), TNF-α (BS2081R, RRID: AB_10856075), Bax (MA5-14003, RRID: AB_10979735), Bcl-2 (MA5-11757, RRID: AB_10978135), Caspase-3 (MA1-91637, RRID: AB_1954999), p53 (DO-7) (MA5-12557, RRID: AB_10989883) and secondary antibodies (as per their compatibility with the primary antibodies); anti-mouse IgG (31430; RRID: AB_228307), anti-rabbit IgG (31460; RRID: AB_228341), anti-mouse Alexa Fluor™ 488 (A-11001; RRID: AB_2534069) were acquired from Thermofisher Scientific (Massachusetts, United states). The remaining analytical-grade reagents used in this experiment were procured from standard chemical suppliers.

### Animal care and hepatocarcinogenesis model

Experimental procedures were conducted according to the policies and procedures formulated by the institutional animal ethics committee *(IAEC/KMC/119/2022)* in accordance with the National Committee for the purpose of control and supervision of experiments on animals (CPSEA), India. Healthy male Wistar rats weighing 250–300 g (8–12 weeks old) were housed in controlled temperature and humidity conditions as per standard animal care guidelines (25 ± 1 °C; 65–80%; 12:12 h light/dark cycle). Animals were provided with a standard pellet diet and water during the study period. All animals were acclimatized to standard laboratory conditions for one week before initiating. Physiological parameters such as body weight, food and water consumption were monitored regularly throughout the study period.

Animals (*n* = 24) were randomly allocated into 4 groups, i.e., CONTROL, BIX, DEN and DEN + BIX groups. CONTROL group animals served as vehicle control and were administered 10% DMSO (in saline) thrice a week throughout the study period. Animals in the BIX group were administered BIX (0.1 mg/kg b.w., dissolved in 10% DMSO, intraperitoneally) thrice a week throughout the study duration. This dosage was selected based on our previous study, in which BIX effectively inhibited early events associated with DEN-induced hepatocarcinogenesis [20]. DEN group animals were administered with *DEN* (50 mg/kg b.w, i.p.) alone weekly once till 14th week of the study period [21]. In the DEN + BIX group, BIX treatment was initiated two weeks prior to DEN injection, continued throughout the 14-week DEN treatment period, and until the end of the study. DEN and BIX were administered as described for the DEN and BIX groups, respectively (ESI Fig. S1).

#### Tumor statistics

After 20 weeks of the treatment period, all animals were sacrificed and analyzed for tumor growth. The liver-to-body weight was computed to determine the hepatosomatic index. Tumors were morphologically analyzed and based on their diameter, categorized as small (< 3 mm) and big (≥ 3 mm) tumors. Tumor incidence was calculated as the proportion of animals that developed at least one visible tumor relative to the total number of animals in the group. Tumor multiplicity was determined by dividing the total number of tumors counted by the total number of tumor-bearing animals.

#### Microscopic examination of hepatic tumors

The histopathological changes in hepatic tumors were assessed by fixing the samples in formalin (10% w/v), followed by paraffin embedding. Hematoxylin and eosin staining of.

Paraffin-embedded Sect. (5 μm) were prepared as per standard laboratory protocol [22]. The processed tissue sections were analyzed for histoarchitectural changes under a conventional optical microscope (Labomed ^®^ Lx 300, California, US).

#### Bioimpedance spectroscopy of hepatic tumors

Bioimpedance spectroscopy was used to assess tumor-associated alterations in the electrical properties of hepatic tumors, as described earlier [23]. Briefly, the bioelectrical conductance of hepatic tissues/tumors was recorded in a frequency range of 4 Hz-5 MHz to capture frequency-dependent electrical properties associated with extracellular matrix characteristics, ion mobility, cell membrane capacitance, and membrane integrity. Thirteen data points per measurement were recorded twice per animal using LCR meter (Hioki-IM3570, Japan). Conductance values were converted to electrical conductivity (σ, S/m) using a predetermined cell constant. Based on conductivity values, tumors were classified as moderate- and high-conducting tumors for subsequent analysis.

#### Quantitative assessment of endoplasmic reticulum stress *via* RT-qPCR

Real-time quantitative PCR (RT-qPCR) was used to assess ER-stress following the method outlined by Xu et al. [24]. Briefly, total RNA was extracted from the liver tissue using the phenol-chloroform method. Optimal concentration of RNA and purity were determined spectrophotometrically using NanoDrop equipment (BioTek Instruments, Winooski, VT, USA). Further, RNA was reverse transcribed to cDNA using the iScript cDNA Synthesis Kit (Bio-Rad Laboratories, Inc., CA, USA). The primer sequences were designed based on data obtained from the National Center for Biotechnology Information (NCBI) and are listed in Table 1. PCR amplification reaction was carried out using SYBR Green and ROX master mix (Thermo Fisher Scientific, Paisley, UK). The amplification parameters of the thermocycler were set to 94 °C for initial denaturation (2 min), 15 s for final denaturation, followed by primer annealing at 60 °C for 30 s (40 cycles), and final elongation at 72 °C for 10 s. Relative mRNA expression was calculated using the 2^−ΔΔCt^ method with GAPDH as the reference gene. The mean ΔCt of the control group served as the calibrator for calculating ΔΔCt values for each biological replicate. Fold-change values were subsequently log₂-transformed for graphical presentation.

**Table 1:** Primer sequence for quantification of mRNA expression of ER-stress-related genes

Gene name	Genebank Accession no.	Primers	Product length (bp)
*EiF2/* *PERK*	NM_031599.2 R	F: CGAAGTGACCGTGGAAGAC R: ATCCCACGTCCAAATCCCAC	198
*Grp-78/* *HSPA5*	NM_013083.2 R	F: TCGGACGCACTTGGAATGAC R: CCAAATACGCCTCGGCAGTT	189
*ATF-6*	NM_001107196.1 R	F: CCAGGTGGTGTCAGAGAACC R: GCAGGGCTCACACTAGGTTT	156
*Ddit3/* *CHOP*	NM_001109986.1 R	F: TACACCACCACACCTGAAAGC R: TCAAAGGCGAAAGGCAGAGA	74

All primers were designed for species-*Rattus norvegicus* using NCBI-Primer-blast-tool

#### Quantitative and qualitative assessment of endoplasmic reticulum stress markers using ELISA and western blotting, respectively

The expression of ER-stress markers PERK and p-PERK was assessed using ELISA as described earlier [25]. Briefly, antigens were extracted from hepatic tumors using Tris-NaCl protein extraction buffer. The extracted antigens (normalized to 1 mg/mL protein) were coated on all ELISA plates (Himedia, India) and incubated at 4 ℃ for 12 h. The antigen-coated plates were then treated with wash buffer (PBSTw) and blocked with a blocking agent for non-specific binding. Further, wells were treated with primary antibodies such as PERK (1:4000) and p-PERK (1:1000) (100 µL) and incubated for 2 h. Following a washing step, the wells were then treated with HRP-conjugated secondary antibody (1:3000 dilution; goat anti-rabbit IgG). The reaction was undertaken at ambient room temperature for 1.5 h, followed by TMB (3,3′,5,5′-Tetramethylbenzidine) addition. 50 µL (2 M) sulfuric acid was then added to the respective wells to stop the reaction. The expression of proteins of interest was quantified by spectrophotometric reading at 420 nm using a multimode microplate reader (BioTek, Synergy H1, Agilent Technologies, USA). The expression of ER-stress marker protein was further quantified using western blotting. Briefly, 40 µg of proteins extracted from the hepatic tumor were separated by 10% sodium dodecyl sulfate–polyacrylamide gel electrophoresis (SDS/PAGE) and transferred to a nitrocellulose membrane. The membranes were then blocked with 5% non-fat dry milk prepared in Tris-buffered saline containing Tween-20 (TBST), washed, and incubated with primary antibody PERK (1:5000) and phospho-PERK (1:5000) & β-Actin/GAPDH (1:10000). The TBST-washed membranes were then probed with HRP-conjugated secondary antibody (HRP-conjugated Goat anti-Rabbit IgG (H + L), 1:2000). Proteins of interest were visualized using Clarity™ western enhanced chemiluminescence (ECL) substrate in ImageQuant LAS 4000 gel documentation system. The levels of expression of each protein were imaged and quantified using ImageJ software.

#### Quantitative assessment of inflammatory, proliferation, tumor suppressor, and apoptotic proteins using enzyme-linked immunosorbent assay

The inflammatory (IL-6, IL-10, TNF-α), proliferation (Ki-67), apoptotic (Bax, Bcl-2, Caspase-3) and tumor suppressor (p-53) proteins expression were quantified using ELISA as described earlier [26]. Briefly, the antigens extracted from the hepatic tumors were coated onto the polystyrene ELISA plates and incubated at 4 ℃. After blocking and washing steps, plates were incubated with primary antibodies (1:500) followed by the appropriate HRP-conjugated secondary antibody (goat anti-rabbit IgG, 1:3000/goat anti-mouse IgG, 1:5000). After the incubation period, the reaction was stopped, and absorbance was read at 420 nm to determine the relative protein expression.

#### Immunohistochemical assessment of proliferation, tumor suppressor, and apoptotic protein markers

Quantitative immunofluorescence analysis was performed as per the method described by Weidensteiner et al. [27]. Briefly, paraffin-embedded sections were dehydrated in xylene and rehydrated through a graded ethanol series. Antigen retrieval was carried out in 10 mM hot sodium citrate buffer (pH 6.0). The tissue sections were then blocked with 10% bovine serum albumin (BSA) for 1 h at room temperature to prevent non-specific binding. Subsequently, the sections were treated with primary antibodies such as Ki-67 (1:200), PCNA (1:200), p53 (1:500), Bax (1:200), Bcl-2 (1:200), Caspase-3 (1:500) and incubated overnight at 4 °C. After incubation, tissue sections were washed three times with phosphate-buffered saline containing 0.1% Tween-20 (PBST) for 5 min each. The sections were then incubated with the appropriate Alexa Fluor 488-conjugated secondary antibody (goat anti-rabbit or goat anti-mouse IgG; 1:1500) for 1.5 h at room temperature in the dark. Following a PBST-wash, tissue sections were counterstained with DAPI (1:1000 in PBS) for 5 min. Further, slides were mounted with an antifade mounting medium and examined under a fluorescence microscope (Axioscope 5, Zeiss, Germany). The percentage of positively stained nuclei was quantified using ImageJ software from randomly selected microscopic fields (3–5) and expressed as the percentage of positive nuclei relative to the total number of DAPI-stained nuclei.

#### Antioxidant defense profile of hepatic tissues/tumors

The hepatic tumor homogenates (10% w/v) were prepared in 0.1 M KH₂PO₄ buffer [28]. Lipid peroxidation (LPO) was estimated as the amount of MDA-TBA chromophore formed/mg protein/minute [29]. Reduced glutathione (GSH) levels were estimated as the amount of 2-nitro-5-mercaptobenzoic acid (TNB) formed/mg protein [30]. Glutathione reductase (GR) was estimated as the amount of NADPH oxidized during the reduction of oxidized glutathione (GSSG)/mg protein/minute. Glutathione peroxidase (GPx) activity was estimated as the amount of NADPH oxidized during the reduction of H_2_O_2_ into H_2_O/mg protein/minute. Superoxide dismutase (SOD) activity was estimated as the amount of enzyme required to scavenge superoxide radicals generated during photo-oxidation of hydroxylamine/mg protein.

Total protein concentration in the sample was estimated by Lowy’s method [31]. Briefly, the assay quantified the amount of cupric protein complex formed when proteins reacted with alkaline copper tartarate and reduced Folin-Ciocalteu to blue molybdate complex. The standard used in this assay was bovine serum albumin.

### Statistical assessments

Results were expressed as mean ± S.D. The Shapiro-Wilk test was applied to assess the data normality. Levene’s test was used to analyze the variance homogeneity. To perform intergroup comparisons and assess the statistical significance, a one-way ANOVA followed by Post hoc Tukey’s test was performed using Jamovi Desktop version 2.6.44 software. The unpaired Student’s t‑test was applied to evaluate the statistical significance of tumor multiplicity. *p* ≤ 0.05 was considered statistically significant.

## Results

### BIX treatment prevented *DEN*-induced hepatocarcinogenesis

The BIX treatment was effective in preventing *DEN*-induced hepatocarcinogenesis. The chemopreventive effect of BIX was observed at a dosage of 0.1 mg/kg b.w. administered intraperitoneally three times a week over 20 weeks. The animals in the DEN group exhibited deteriorated health, as evidenced by significantly *(p* ≤ 0.05) decreased body mass accompanied with decreased food & water intake as compared to CONTROL groups (ESI Fig. S2a). However, BIX treatment improved the overall health of animals as indicated by significantly (*p* ≤ 0.05) increased body mass, food & diet intake as compared to DEN group (ESI Fig. S2b & c).

**Fig. 1:**
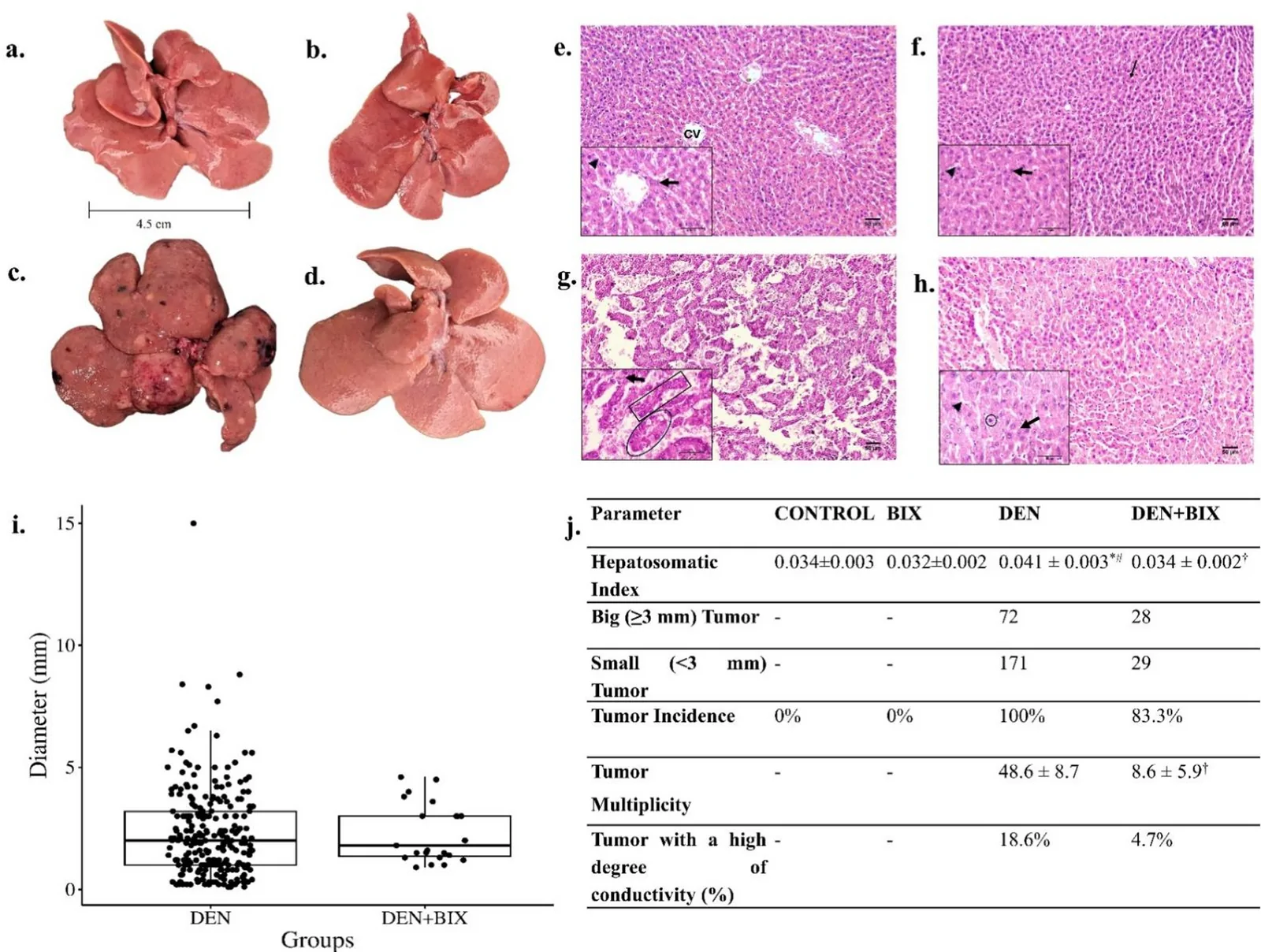
Macroscopic morphology and histopathological examination of hepatic tissues/tumors **a** & **b** represents CONTROL and BIX groups showing preserved hepatic lobular structure with distinct lobes of the liver. **c** represents the DEN group showing malformed, stiffened, and enlarged lobes with multiple tumor nodules. **d** DEN + BIX group depicting smooth hepatic surfaces and fewer tumor nodules. **e** & **f** Photomicrographs of CONTROL and BIX group liver showing normal hepatic histoarchitecture with hepatocytes radiating from the central vein and preserved sinusoidal space (arrow showing hepatocytes and arrowhead showing sinusoidal space; in inset). **g** DEN group showing pseudoglandular (acinar) architectural pattern (encircled area; in inset), with focal thickened trabeculae (boxed area; in inset), and nuclear atypia (arrow; inset) indicating moderately differentiated HCC. **h** DEN + BIX group showing preserved hepatic architecture with restoration of hepatocyte plates and sinusoidal spaces (arrow and arrowhead; inset), and minimal cytologic atypia (circle; inset), indicating a well-differentiated stage of HCC. **i** represents the overall tumor size distribution in DEN and DEN + BIX treated groups. **j** Effect of BIX treatment on tumor statistics (The hepatosomatic index was statistically evaluated using one-way ANOVA followed by Tukey’s HSD. The statistical significance of tumor multiplicity was evaluated using Student’s *t*-test. ^*^ represents statistical significance (*p* ≤ 0.05) w.r.t CONTROL, ^#^ represents statistical significance (*p* ≤ 0.05) w.r.t BIX, and ^†^ represents statistical significance *(p* ≤ 0.05) w.r.t DEN group; Magnification: 100×; Inset image magnification: 400× with scale bar 50 μm)

The gross morphological analysis of liver tissues from DEN and DEN + BIX treatment groups demonstrated enlarged hepatic lobular structures with distortion and morphological alterations as compared to CONTROL and BIX groups (Fig. 1). The total hepatic tumor count in DEN and DEN + BIX groups were 243 and 57, respectively. Tumor incidence was 100% and 83.3% in DEN and DEN + BIX groups, respectively. Tumor multiplicity was significantly (*p* ≤ 0.05) decreased in DEN + BIX group in comparison to the DEN group (Fig. 1j). Further, histopathological evaluation indicated that most of the tumors in DEN group were moderately differentiated, exhibiting predominantly pseudoglandular (acinar) architectural patterns with focal thickened trabeculae and nuclear atypia with increased nuclear-to-cytoplasmic ratios. However, BIX treatment effectively inhibited the progression of hepatocarcinogenesis, with most tumors at the well-differentiated stage of hepatocarcinogenesis (Fig. 1e-h). These findings were further supported by the dielectric properties of tumors, where DEN group exhibited 18.76% high conducting tumors when compared with 4.7% in DEN + BIX group.

### BIX treatment modulated the evolution of hepatic tumors

**Fig. 2:**
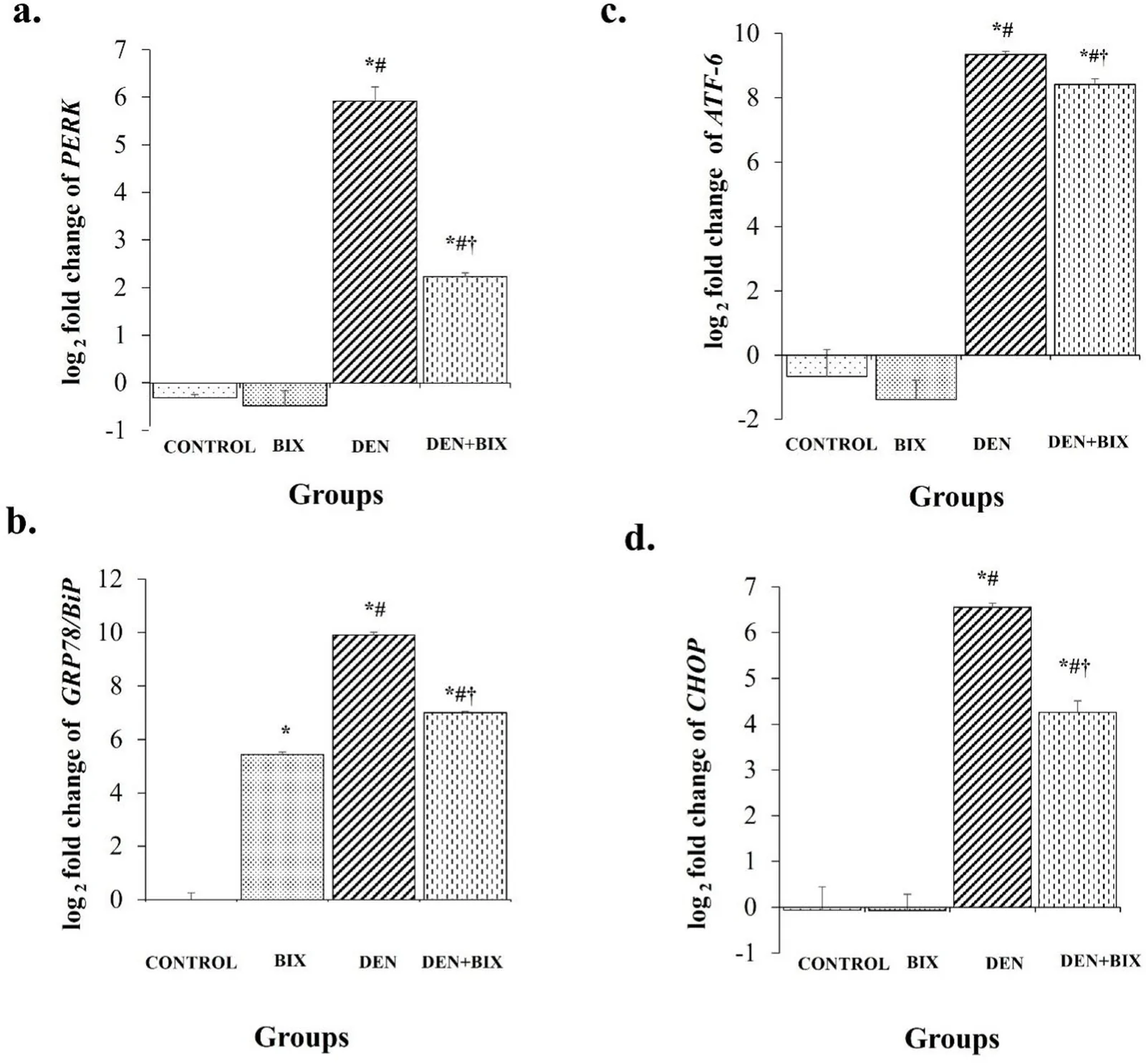
Effect of BIX on UPR gene expression as determined by RT-qPCR. **a** Effect of BIX on the expression of *PERK* gene in different groups as determined by RT-qPCR. **b** Effect of BIX on the expression of *GRP78/BiP* gene in different groups as determined by RT-qPCR. **c** Effect of BIX on the expression of *ATF-6* gene in different groups as determined by RT-qPCR. **d** Effect of BIX on the expression of *CHOP* gene in different groups as determined by RT-qPCR. Relative mRNA expression was normalized to *GAPDH* using the 2^−ΔΔCt^ method and is presented as log_2_ fold change relative to the control group. (Data was expressed as mean ± S.D and analyzed using one-way ANOVA followed by post hoc test (Tukey’s HSD). ^*^ represents significance (*p* ≤ 0.05) w.r.t CONTROL group; ^#^ represents significance (*p* ≤ 0.05) w.r.t to BIX group; ^†^ represents significance (*p* ≤ 0.05) w.r.t to DEN group)

**Fig. 3:**
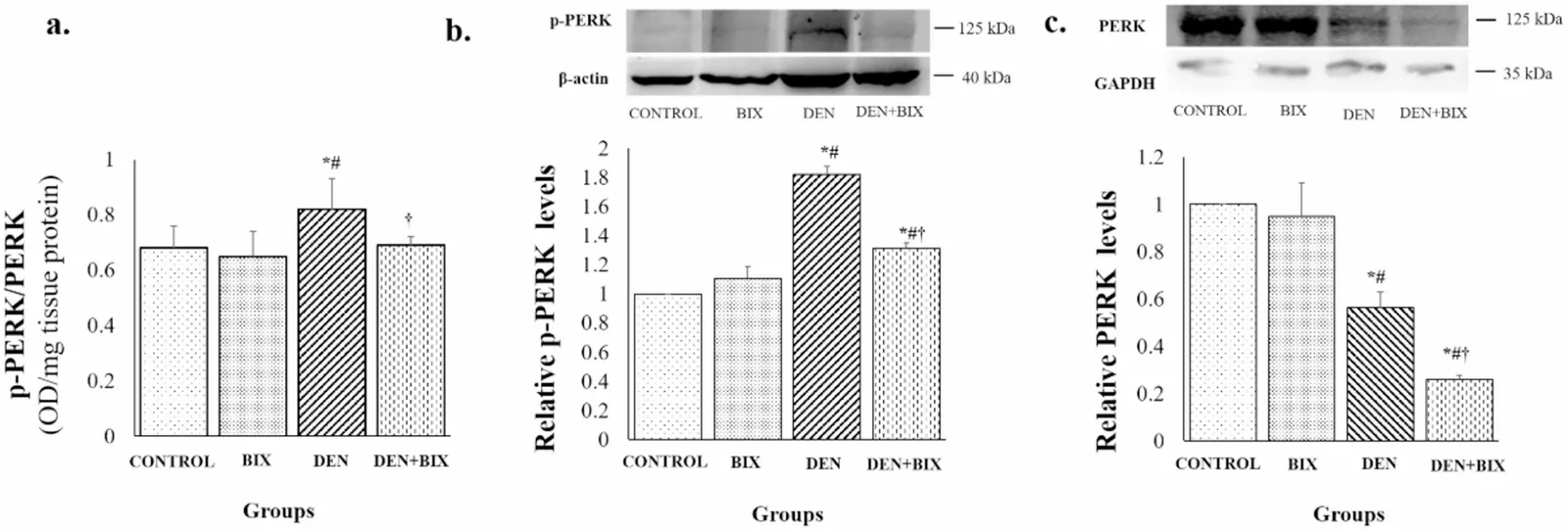
Effect of BIX on p-PERK and PERK protein expression as determined by ELISA and western blot. **a** Effect of BIX on the protein expression of p-PERK/PERK ratio across different groups as determined by ELISA. b & **c** Effect of BIX on protein expression of p-PERK and PERK across different groups as determined by western blot (representative blot shown) and densitometric analysis of band intensity. β-actin/GAPDH was used as an internal loading control. (Data was expressed as mean ± S.D and analyzed using one-way ANOVA followed by post hoc test (Tukey’s HSD). ^*^ represents significance (*p* ≤ 0.05) w.r.t CONTROL group; ^#^ represents significance (*p* ≤ 0.05) w.r.t to BIX group; ^†^ represents significance (*p* ≤ 0.05) w.r.t to DEN group)

Persistent ER stress plays a crucial role in the evolution of cancer. Therefore, the upregulated unfolded protein response (UPR) is one of the hallmarks of hepatocarcinogenesis [32]. RT-qPCR analysis of the *DEN*-treated group demonstrated significantly (*p* ≤ 0.05) increased gene expression of UPR components, *PERK* and *ATF-6*. However, the DEN + BIX treated group demonstrated significantly (*p* ≤ 0.05) decreased *PERK* and *ATF-6* gene expression as compared to DEN group (Fig. 2a & c). These findings were further supported by western blotting and ELISA protein expression, which showed a significant (*p* ≤ 0.05) decrease in p-PERK/PERK ratio in DEN + BIX group as compared to DEN group (Fig. 3).

In addition, RT-qPCR analysis also revealed that BIX treatment successfully induced *GRP78* expression, as indicated by significantly (*p* ≤ 0.05) increased *GRP78* gene expression in the BIX-treated group as compared to CONTROL group. *GRP78* gene expression was also significantly (*p* ≤ 0.05) increased in DEN and DEN + BIX groups as compared to CONTROL group (Fig. 2b). Further, *CHOP* (C/EBP Homologous Protein) gene expression was also significantly (*p* ≤ 0.05) decreased in DEN + BIX group as compared to DEN group (Fig. 2d).

### BIX treatment attenuated *DEN*-induced inflammation and tumor cell proliferation in hepatic tumors

**Fig. 4:**
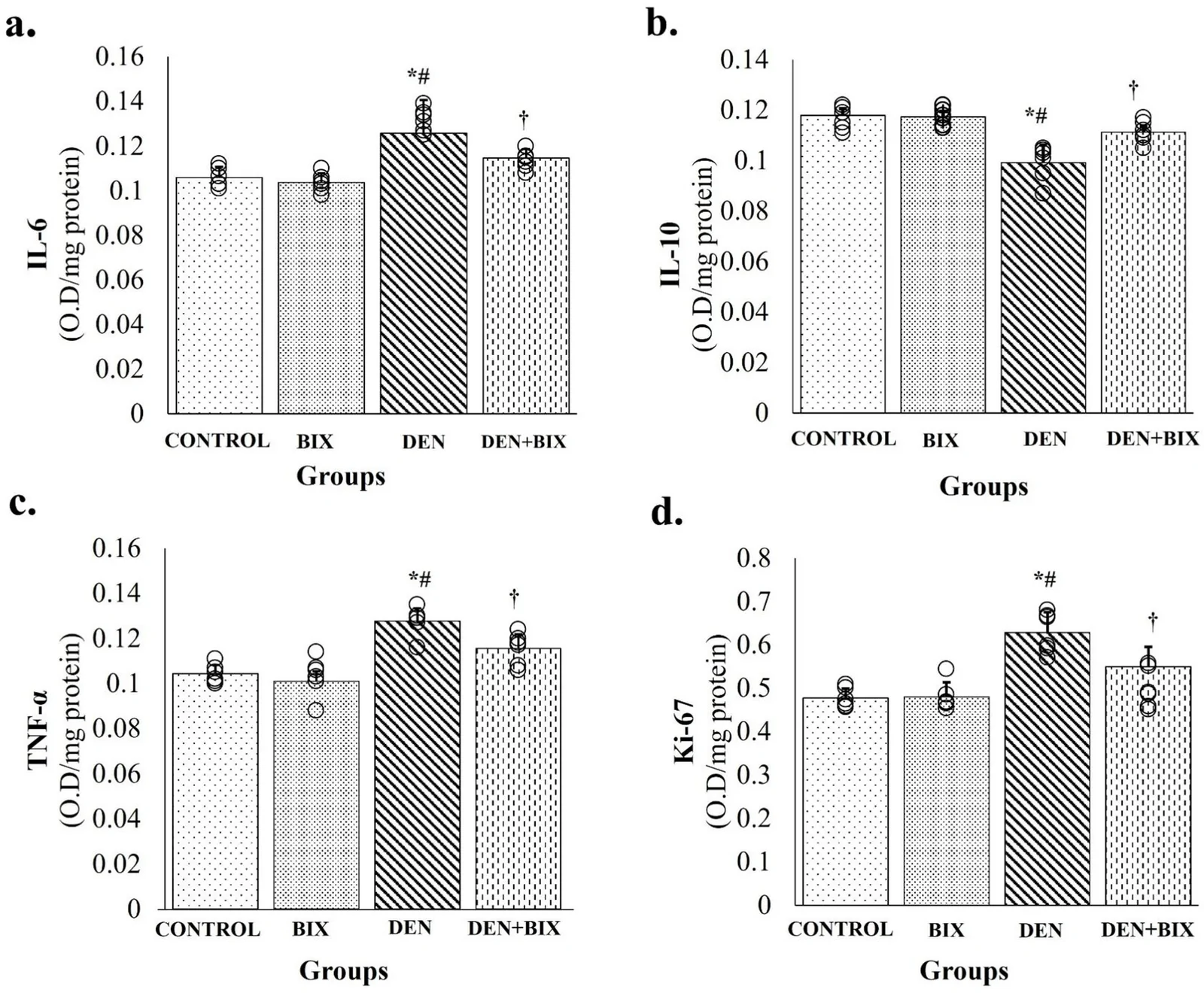
Effect of BIX on inflammatory cytokines and proliferation marker Ki-67 in hepatic tumors using ELISA. **a** IL-6 expression across different treatment groups. **b** IL-10 expression across different treatment groups. **c** TNF-α expression across different treatment groups. **d** Ki-67 expression across different treatment groups. (Data were expressed as mean ± S.D (*n* = 6) and analyzed using one-way ANOVA followed by post hoc test (Tukey’s HSD). ^*^ represents significance (*p* ≤ 0.05) w.r.t CONTROL group; ^#^ represents significance (*p* ≤ 0.05) w.r.t to BIX group; ^†^ represents significance (*p* ≤ 0.05) w.r.t to DEN group)

**Fig. 5:**
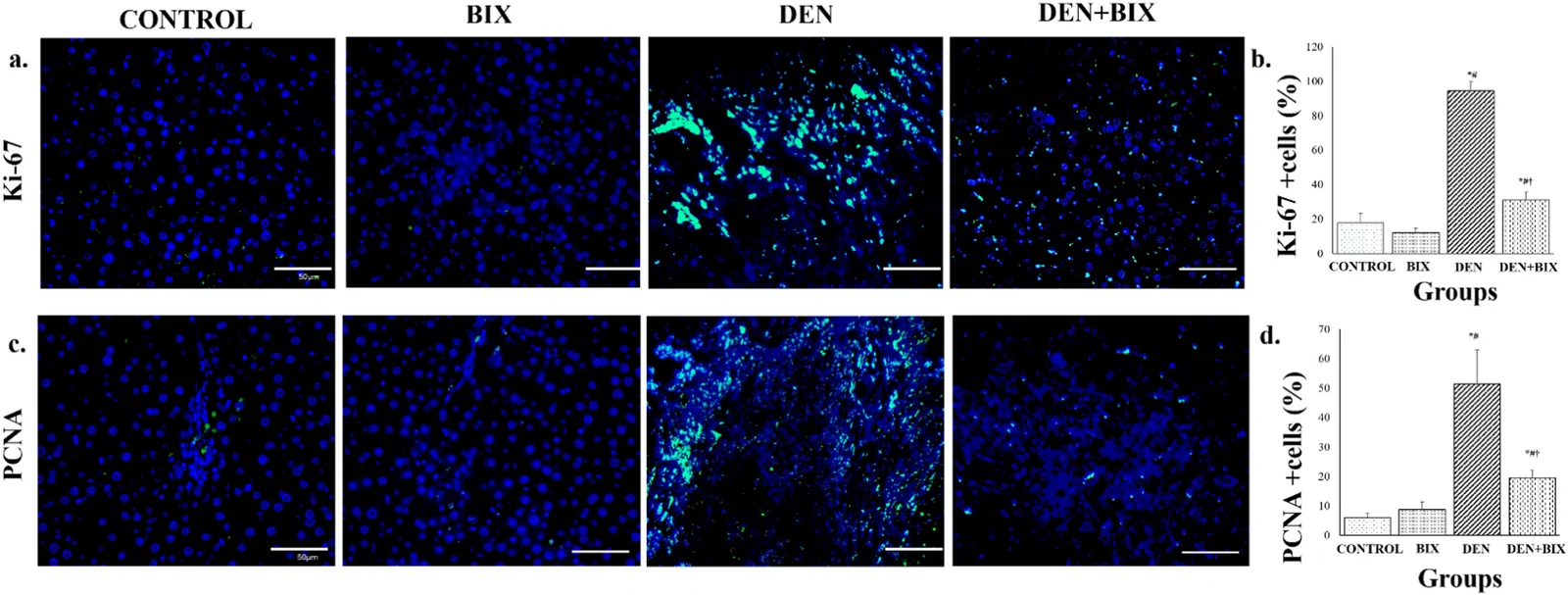
Effect of BIX on Ki-67 and PCNA in hepatic tumors using fluorescent immunohistochemical staining. **a** Representative immunofluorescence images showing Ki-67 (green) and DAPI (blue) staining in different treatment groups. **b** Ki-67-positive cell percentage using ImageJ. **c** Representative immunofluorescence images showing PCNA (green) and DAPI (blue) staining in different treatment groups. **d** PCNA-positive cell percentage using ImageJ. Magnification: 400×; scale bar: 50 μm. (Data were expressed as mean ± S.D and analyzed using one-way ANOVA followed by post hoc test (Tukey’s HSD). ^*^ represents significance (*p* ≤ 0.05) w.r.t CONTROL group; ^#^ represents significance (*p* ≤ 0.05) w.r.t to BIX group; ^†^ represents significance (*p* ≤ 0.05) w.r.t to DEN group)

The chronic inflammatory processes are often associated with *DEN*-induced hepatocarcinogenesis, where pro-inflammatory mediators like interleukins (ILs) and TNF-α activate tumor-promoting pathways that drive both inflammation and cell proliferation. The current findings showed that BIX treatment significantly (*p* ≤ 0.05) decreased the expression of inflammatory cytokines (IL-6 and TNF-α) in DEN + BIX group as compared to the DEN group. In contrast, anti-inflammatory cytokine IL-10 was significantly (*p* ≤ 0.05) increased in DEN + BIX group as compared to the DEN group (Fig. 4a-c). To further assess the tumor proliferative activity, Ki-67 and PCNA fluorescent immunohistochemical analyses were performed. The DEN-treated group exhibited increased Ki-67 and PCNA immunoreactivity with a significantly higher percentage of positively stained cells as compared to the CONTROL group, indicating enhanced cellular proliferation. In contrast, BIX treatment significantly (*p ≤ 0.05)* decreased Ki-67 and PCNA expression as compared with the DEN group (Fig. 5a & c). These findings were further supported by ELISA, which demonstrated a significant reduction in proliferative marker Ki-67 expression following BIX treatment. Collectively, these results suggested that BIX treatment suppressed DEN-induced hepatic tumor growth by attenuating both inflammatory signaling and tumor cell proliferation.

### BIX treatment prevented *DEN*-induced suppression of p53 and apoptosis regulators

**Fig. 6:**
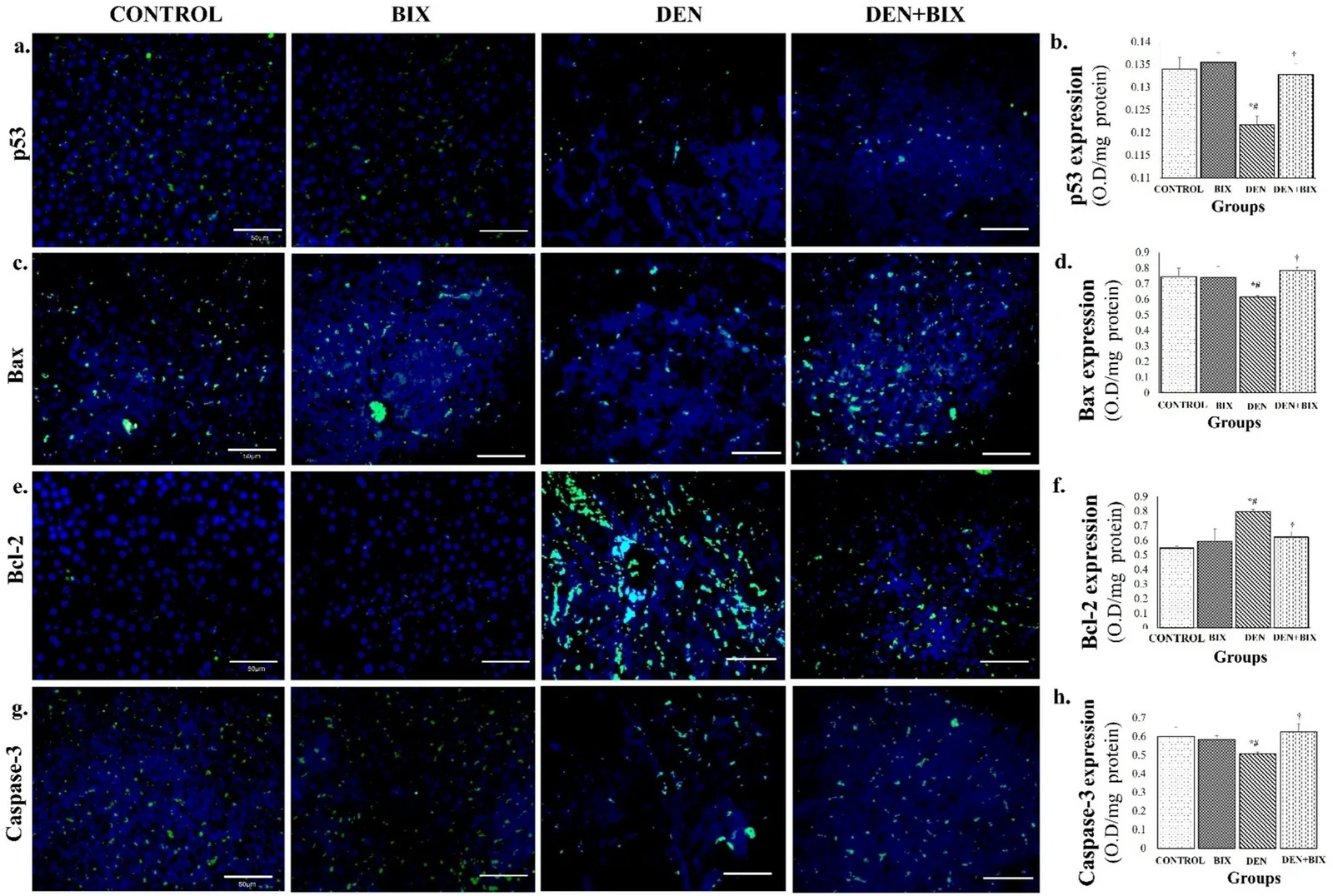
Effect of BIX on the expression of p53 and apoptosis signaling proteins. **a** & **b** Representative immunofluorescence micrographs and corresponding quantitative ELISA analysis of p53 protein expression in the different experimental groups. **c** & **d** Representative immunofluorescence micrographs and corresponding quantitative ELISA analysis of Bax protein expression. **e** & **f** Representative immunofluorescence micrographs and corresponding quantitative ELISA analysis of Bcl-2 protein expression. **g** & **h** Representative immunofluorescence micrographs and corresponding quantitative ELISA analysis of cleaved caspase-3 protein expression. Expression of related proteins was visualized as green fluorescence, while nuclei were counterstained with DAPI (blue). Magnification: 400×; scale bar: 50 μm. (Data were expressed as mean ± S.D and analyzed using one-way ANOVA followed by post hoc test (Tukey’s HSD). ^*^ represents significance (*p* ≤ 0.05) w.r.t CONTROL group; ^#^ represents significance (*p* ≤ 0.05) w.r.t to BIX group; ^†^ represents significance (*p* ≤ 0.05) w.r.t to DEN group)

DEN-induced modulation of the p53-mediated apoptotic pathway is a key characteristic feature of hepatocarcinogenesis. The results of the present study showed that BIX treatment demonstrated significantly (*p* ≤ 0.05) increased p53 expression in DEN + BIX group as compared with DEN group (Fig. 6b). Further, DEN + BIX group showed significantly (*p* ≤ 0.05) increased Bax and Caspase-3 expression as compared to DEN group (Fig. 6d & h). In contrast, expression of anti-apoptotic protein Bcl-2 was significantly (*p* ≤ 0.05) decreased in DEN + BIX group when compared to DEN group (Fig. 6f). These results were further supported by fluorescent immunohistochemistry.

### BIX treatment strengthened endogenous antioxidant capacity in *DEN*-exposed animals

BIX treatment significantly (*p* ≤ 0.05) decreased cellular ROS levels by 1.43-fold in DEN + BIX group when compared with DEN group. Lipid peroxidation was significantly (*p* ≤ 0.05) decreased in DEN + BIX group when compared to DEN group. Further, BIX administration significantly (*p* ≤ 0.05) decreased oxidative stress markers and improved the antioxidant balance by enhancing the activity of enzymatic antioxidants (SOD, GPx) and non-enzymatic antioxidants (GSH) by 1.37, 1.07 and 1.31 folds, respectively as compared with DEN group (Table 2).

**Table 2:** Effect of BIX in modulating antioxidant defense system

Parameter	CONTROL	BIX	DEN	DEN + BIX
LPO (nmol/min/mg protein)	0.94 ± 0.02	0.98 ± 0.06	1.42 ± 0.01^*#^	0.96 ± 0.01^†^
Reduced GSH (nmol/mg protein)	3.83 ± 0.10	4.035 ± 0.23	2.72 ± 0.13^*#^	3.71 ± 0.21^†^
GR (nmol/min/mg protein)	1.06 ± 0.12	1.12 ± 0.06	0.80 ± 0.01^*#^	1.03 ± 0.02^†^
GPx (nmol/min/mg protein)	1.11 ± 0.03	1.07 ± 0.02	0.70 ± 0.06^*#^	0.91 ± 0.02^*#†^
SOD (IU/min/mg protein	0.19 ± 0.01	0.19 ± 0.02	0.13 ± 0.01^*#^	0.17 ± 0.01^†^
ROS (Relative intensity AFU)	237.09 ± 1.40	235.89 ± 2.65	465.47 ± 5.64^*#^	324.87 ± 4.33^*#†^

Data was expressed as mean ± S.D and analyzed using one-way ANOVA followed by post hoc test (Tukey’s HSD)

^*^ represents significance (*p* ≤ 0.05) w.r.t CONTROL group; ^#^ represents significance (*p* ≤ 0.05) w.r.t to BIX group; ^†^ represents significance (*p* ≤ 0.05) w.r.t to DEN group)

## Discussion

Cancer is a complex evolutionary process in which normal cells over a period of time, acquire a spectrum of characteristic hallmarks that eventually drive their transformation into malignant phenotypes [33]. This transformation involves not only genetic mutations and nuclear alterations but also causes significant changes in various cellular organelles. One such organelle is the endoplasmic reticulum, which is known to play a pivotal function in supporting the cellular adaptations necessary for cancer development and progression [34]. Thus, targeting such maladaptive cellular processes has emerged as a new strategy in cancer prevention and therapy [35]. In this study, we evaluated the chemopreventive potential of targeting ER stress for the prevention of hepatocellular carcinoma.

The Hepatocarcinogenesis model was developed using the chemical carcinogen *Diethylnitrosamine* (*DEN*). *DEN* is a procarcinogen that gets activated by the enzyme CYP2E1 located in the ER [36]. The activation of *DEN* leads to the generation of reactive diazonium ions, followed by the formation of highly reactive oxidative radicals, triggering oxidative stress in cells [37]. The cellular oxidative stress is known as a critical mediator of DNA damage and genetic mutations, marking the initiation stage of carcinogenesis [38]. This sustained oxidative stress not only induces genomic instability but also affects organellar genomes and their membrane integrity, thereby impairing the functioning of different cell organelles, including the ER [39]. Eventually, these cells acquire rapid division capacity, which exerts increased demand on the endoplasmic reticulum to upregulate protein synthesis and folding [40].

The elevated cellular demand for synthesis and processing puts the ER under stress and hampers its functioning [41]. Under such conditions, ER restores its function by activating a feedback reaction termed as UPR [42]. However, when ER-stress becomes prolonged and demand for protein folding remains persistently high, the adaptive capacity of UPR becomes insufficient, leading to sustained ER-stress [43]. The hepatic tumors obtained in the present study also demonstrated elevated ER-stress compared to normal tissue as indicated by markers like *PERK* and *ATF-6*. During normal conditions, the ER-stress markers PERK, IRE1, and ATF6 remain in an inactive state by binding to ER chaperone GRP78/BiP [44]. However, during carcinogenesis, since demand for protein synthesis is very high the UPR remains constantly activated, cancer cells adapt or evolve to handle a certain degree of UPR and utilize it to favor their growth and development [45].

Several studies have reported that ER-stress inhibitors, especially chemical chaperones like tauroursodeoxycholic acid (TUDCA), 4-phenylbutyric acid (4-PBA), ursodeoxycholic acid (UDCA) and trimethylamine N-oxide (TMAO), etc., have been widely evaluated for their ability to attenuate ER stress [46–49]. These chemical chaperones have demonstrated a significant inhibitory effect on ER-stress by binding to the misfolded or unfolded ends, thereby preventing aggregation and promoting protein folding [50]. There are mainly two types of strategies to mitigate ER-stress that include the administration of exogenous chemical chaperones and the induction of endogenous molecular chaperones. Among these, the latter approach might be considered a reliable strategy for cancer chemoprevention because endogenous chemical chaperones might facilitate the activation of adaptive UPR signalling for ER homeostasis, unlike exogenous chemical chaperones, which provide external support for UPR alleviation.

BIX (1-(3,4-dihydroxy phenyl)-2-thiocyanato-ethanone), an endogenous chaperone inducer, selectively upregulates the expression of ER resident chaperone gene *GRP78* [15, 19]. It has been reported as a promising ER-stress inhibitor that upregulated BiP mRNA and protein expression, thereby inhibiting the phosphorylation of PERK and eIF2a as observed in several ER stress-induced disease models like cerebral ischemia, neuronal death, rheumatoid arthritis and cardiomyopathy [16, 51–53]. Similar results were noted in our study, where *GRP78/BiP* expression was significantly increased in the BIX-treated group as compared to the untreated CONTROL group. However, *GRP78/BiP* expression in the DEN + BIX group was significantly decreased as compared to the DEN group. This could likely be attributed to the adaptive mechanism of BIX, which might have enhanced the ER protein-folding capacity and restored ER homeostasis during the early stages of hepatocarcinogenesis. Consequently, the burden of unfolded proteins and ER stress may have been alleviated, thereby reducing the need for compensatory *GRP78* overexpression in the DEN + BIX group. This was further supported by the reduced activation of the PERK pathway, as evidenced by the lower p-PERK/PERK ratio together with the downregulation of *PERK*, *ATF6*, and *CHOP* gene expression. Overall, these results suggested that BIX enhanced the levels of GRP78 that might have resolved the ER-stress, thereby dampening the evolution of these tumors. The suppression of tumors by BIX was evidenced by a significantly decreased total tumor count and tumor multiplicity after 20 weeks of BIX treatment. This was further supported by histopathological analysis, where the BIX-treated group demonstrated lower-grade tumors as compared to the untreated DEN group. These observations were further supported by bioimpedance spectroscopy, which was employed as an alternative complementary technique to validate the effect of BIX on tumor growth. The advanced hepatic tumors usually exhibit higher conductivity because of the extensive necrosis and angiogenesis within the tumor tissue [23]. Accordingly, the significantly decreased percentage of highly conductive hepatic tumors in the DEN + BIX group indicated that BIX was effective in impeding the progression of hepatocarcinogenesis.

The chemopreventive potential of BIX might be attributed to its ability to prevent sustained low-level and prolonged activation of ER, which is known to contribute to the progression of hepatocarcinogenesis. During cancer development, the maladaptive activation of PERK-eIF2α, IRE1α–XBP1s and ATF-6 further leads to activation of downstream signaling cascades supporting tumor growth and survival [40]. For example, PERK activation leads to evasion of apoptosis *via* p-eIF2α/ATF4 pathway, resulting in enhanced antioxidant responses and suppressed apoptosis [54]. Similarly, prolonged low-level activation of IRE1α results in activation of TRAF2/ASK1/JNK, thereby stimulating inflammation and proliferation, whereas activated ATF-6 drives transcription of genes that promote cell survival and immune evasion [55]. Similarly, our findings demonstrated that the untreated DEN group exhibited a significant upregulation of anti-apoptotic protein Bcl-2, inflammatory mediators (IL-6 and TNF-α) and proliferation markers (Ki-67 & PCNA) in comparison to the CONTROL group, demonstrating that ER stress might induce oncogenic transformations. However, BIX treatment significantly decreased the expression of Bcl-2, IL-6, TNF-α, Ki-67 and PCNA as compared to DEN group. In addition, BIX treatment simultaneously activated protective signaling that resulted in significantly increased expression of anti-inflammatory (IL-10), pro-apoptotic (Bax) and tumor suppressor (p53) proteins in comparison to the DEN group. These findings clearly indicated that BIX treatment attenuated ER-stress, regulated the balance between anti- and pro-apoptotic proteins, resulting in caspase-dependent apoptotic cell death (ESI Fig. S3).

Overall, the findings from this study suggested that impaired endoplasmic reticulum function plays a crucial role in the initiation and progression of oncogenesis. These findings were consistent with previous studies demonstrating that pharmacological modulation of ER stress suppresses HCC development [56]. For instance, BGP-15-mediated inhibition of ER stress attenuated NASH progression and reduced the incidence of HCC in a high-fat diet-induced MUP-uPA HCC mouse model [57]. Similarly, in another study, PERK inhibitor GSK2656157 for four weeks after 25 weeks of DEN administration significantly decreased tumor burden in the DEN-induced HCC mouse model [32]. Therefore, it can be suggested that targeting ER to restore homeostasis by preventing maladaptive unfolded protein response (UPR) activation can be a promising and effective strategy to impede cancer evolution. As per the best of our knowledge, this is the first study where an endogenous ER chaperone inducer has been employed for cancer chemoprevention. The encouraging results of this study clearly suggested that ER-targeted therapy could be a potential alternative strategy for cancer prevention.

Despite the encouraging findings of the present study, several limitations should be acknowledged. Although the present study demonstrated the involvement of ER stress-associated pathways in hepatocarcinogenesis and their modulation by BIX, the precise molecular mechanisms underlying the protective effects of BIX remain to be elucidated. Therefore, future investigations incorporating in vitro validation, additional protein validation, and assessment of cleaved Caspase-3 would provide a more comprehensive understanding of the molecular mechanisms underlying the protective effects of BIX during hepatocarcinogenesis.

## Supplementary Information

Below is the link to the electronic supplementary material.

Supplementary Material 1

## Data Availability

Data supporting the study can be provided upon a request to the corresponding author.
