# Supplementary material for: Inhibiting endoplasmic reticulum stress and unfolded protein response: an effective strategy for chemoprevention of hepatocarcinogenesis
Source: Mol Cell Biochem. 2026 Aug 24;481(9):4949–62. doi: 10.1007/s11010-026-05708-6 (PMC13638761; doi:10.1007/s11010-026-05708-6)
Supplement: Supplementary file 1 [file 11010_2026_5708_MOESM1_ESM.docx]

**Supplementary information**

**S1. Experimental design and dosing regimen**

**Figure S1. Schematic representation of the experimental design and dosing regimen for evaluating the chemopreventive effect of BIX in DEN-induced hepatocarcinogenesis**

**S.2.** **Physiological observations**

**Figure S2.** **Physiological observations** (a) Body weight of animals at weekly intervals (b) Diet intake of animals at weekly intervals (c) Water intake of animals at weekly intervals.

**S3. Mechanism of action of BiP inducer X (BIX) in the prevention of cancer progressi**on.

**Figure S3. Schematic illustration of the probable mechanism of action of BiP inducer X (BIX) in the prevention of cancer progression.** The figure shows the action of BIX in inhibiting maladaptive activation of UPR (PERK-eIF2α, IRE1α–XBP1s and ATF-6), by selectively inducing BiP gene and ER resident chaperone GRP78/BiP expression to alleviate ER-stress.
